# Relationship between Sports Practice, Physical and Mental Health and Anxiety–Depressive Symptomatology in the Spanish Prison Population

**DOI:** 10.3390/healthcare11060789

**Published:** 2023-03-07

**Authors:** María Penado Abilleira, María-Paula Ríos-de-Deus, David Tomé-Lourido, María-Luisa Rodicio-García, María-José Mosquera-González, Daniel López-López, Juan Gómez-Salgado

**Affiliations:** 1 Facultad de Ciencias de la Salud, Universidad Internacional de la Rioja, 26004 Logroño, Spain; 2 Department of Specific Didactics, Research, and Diagnose Methods, Grupo de Investigación FORVI (Formación y Orientación para la Vida), Universidade da Coruña, 15071 A Coruña, Spain; 3 Department of Psychology, Universidade da Coruña, 15071 A Coruña, Spain; 4 Department of Physical and Sport Education, Grupo de Investigación FORVI (Formación y Orientación para la Vida), Universidade da Coruña, 15179 A Coruña, Spain; 5 Research, Health and Podiatry Group, Department of Health Sciences, Faculty of Nursing and Podiatry, Industrial Campus of Ferrol, Universidade da Coruña, 15403 Ferrol, Spain; 6 Department of Sociology, Social Work and Public Health, Universidad de Huelva, 21071 Huelva, Spain; 7 Safety and Health Postgraduate Programme, Universidad Espíritu Santo, Guayaquil 092301, Ecuador

**Keywords:** health, sports practice, anxiety, depression, prisons, inmate, wellness

## Abstract

The objective of this study was to evaluate, in a group of Galician inmates, if there were variations in the levels of physical and mental health and anxiety–depressive symptomatology, depending on the sports’ practice performed. The relationship between these constructs was also investigated. The sample was composed of 509 people deprived of liberty in prisons in the Autonomous Community of Galicia, Spain. A quantitative methodology was used, with the questionnaire as an information collection instrument, Student’s *t*-tests, Pearson’s correlation analysis and a stepwise regression analysis were carried out. The results indicated that those who performed physical activity during their stay in prison had higher levels of physical and mental health, as well as lower indicators of anxiety–depressive symptoms. People who did not practice sports showed a decrease in their perceived health levels when compared to those perceived in their pre-prison stage. A negative association was shown between perceived health levels and anxiety–depressive symptomatology. Perceived physical health, alone, explained 35% of the variance in perceived mental health. These results add to knowledge about the relationships between perceived health, anxiety–depressive symptoms and sports practice in the group of people deprived of liberty, highlighting the importance of promoting physical activity in penitentiary institutions.

## 1. Introduction

Throughout the last few decades, the scientific literature has collected abundant evidence about the benefits that the practice of physical and sports activities have for people’s health [1]. In terms of physical health, regular physical activity is effective in preventing at least twenty-five chronic medical conditions, with a risk reduction that ranges from 20 to 30% [2,3,4]. The mere fact that people become a little more active in their day to day lives, without reaching the recommendations of one hundred and fifty minutes of physical activity per week from the different international agencies, already implies health benefits [1]. This improvement in health, caused by physical activity, is independent of educational or economic levels [5].

In the same vein, there are many scientific publications that reflect the importance of exercise in adults: not only to improve physical health levels, but also regarding mental health levels [6,7]. Specifically, a wide range of positive results have been identified, regarding sports participation in relation to social, psychological and psychosocial health, such as perceived social support, the sense of belonging or a greater self-esteem [6]. These health benefits that derive from the practice of physical activity and sports have also been found in other age groups, such as in children [8], adolescents [9] or the elderly [10].

Regarding the prison population, inmates usually have worse levels of physical and mental health, tending to suffer from chronic physiological and psychological disorders [11]. In terms of physical health, inmates are disproportionately affected by risk factors for cardiovascular diseases [12] and tend to be obese through rapid weight gain during their stay in prison [13,14].

However, different investigations have shown that structured physical activity can improve cardiovascular risk levels and obesity during hospitalization [14,15]. Similarly, it has also been proven that the practice of physical activity in prison improves the well-being perceived by inmates as well as reducing their levels of depression. The promotion of physical activity is also established as a critical component in older inmates when maintaining a good lifestyle in prison, preventing the onset of the previously mentioned diseases [16].

On the other hand, in relation to mental health, although prison inmates tend to come from socially disadvantaged environments, presenting poor health indicators linked to their situations of social exclusion, detention centers may offer an opportunity to combat these inequalities through health promotion programs [17]. In the international context, there are numerous interventions based on sport and physical activity carried out in prisons with the purpose of improving the levels of inmates’ psychological well-being and reducing mental symptoms [4,18,19,20]. In general, these interventions usually last between six weeks and nine months, and as a general conclusion, it has been established that they provide a positive impact on the psychological well-being of inmates [20]. Physical activity in prison leads to reduced despair among inmates [21], becoming a coping strategy to deal with incarceration and decrease levels of anxiety and depression [22].

As for the prison population of women, the benefits of physical activity are the same as for men in terms of physical and mental health, allowing them to develop a positive social identity and favoring social relationships [3,23,24]. However, the participation of inmates in these activities is very low compared to the levels of physical exercise performed by men, or those recommended for society in general, due to the many obstacles that exist when it comes to participating in physical activities in prison [25]. Among these obstacles, the institutional system itself stands out: the internal functioning, family demands or the need to work in prison, lead to limited time available for sports [26].

In the Spanish context, over the years, legislation has evolved and been adapted to conceive of sports as a habitual activity in prison, aimed at achieving the re-education and social reintegration of inmates [27,28,29]. Therefore, over the last few years, different projects have been carried out implementing proposals to promote physical activities and sports in prisons [30].

The purpose of these programs is to enhance the cardiovascular health of inmates [31], establish a health education system [32], develop maps of assets for health in young inmates [33], learn about the role of sports in prison for social rehabilitation [34], assess the satisfaction and perception of this population with sports programs that seek to promote their reintegration [35,36] or promote the values of responsibility, commitment and enthusiasm [37].

The review of the existing literature reveals the interest in the subject and that more and more there is a tendency to work with specific groups within the prison. It will be necessary to look for references that make it possible to compare what happens in different institutions, since each of them has its own operating dynamics and this can condition moods to the point of wanting to go to prison, as was stated by some inmates, recidivists, in order to have a more orderly, controlled and safe life. A novelty compared with previous studies is having used the sports practice variable prior to entering prison to see to what extent it provided relevant information to the works published to date, in addition to providing data from the Autonomous Community of Galicia.

The objective of this research was to analyze whether there are variations in the levels of physical health, mental health and anxiety–depressive symptomatology, in people who were inmates in Galician prisons, depending on the sports practice carried out before and after their entry into prison and above all, we wanted to inquire about the relationships between physical health, mental health and anxiety–depressive symptoms during the duration of the sentence.

## 2. Method

### 2.1. Participants

The sample was made up of 509 participants (88.2% men and 11.8% women) interned in penitentiary institutions in the Autonomous Community of Galicia. The ages ranged between 19 and 74 years (M = 40.85; SD = 10.55), with women being slightly younger than men, but without these differences being considered significant.

Regarding criminal variables, men and women presented differential traits with respect to whether it was the first time they were imprisoned or, on the other hand, whether they were repeat offenders and if the sentence imposed contemplated the commission of a single crime or several (x^2^ = 343.365, *p* < 0.01). A greater recidivism and greater probability of committing multiple crimes were observed in male inmates (see Table 1).

For the classification of the crimes committed by the inmates, the International Classification of Crime for Statistical Purpose (UNODC, 2015) provides the following categories of Level 1 crimes (see Table 2).

In addition to the previous categories, an extra category (number 12) was established that includes the commission of crimes against a partner, ex-partner or person linked with the same bond or emotional relationship by a man and that constitutes a specific typology of the Spanish judicial environment called gender violence.

Regarding the type of crime committed by men and women, the most common in both genders were crimes against property that involved the use of violence (theft and robbery) followed by crimes against public health or that are considered acts with psychoactive substances (drug trafficking). Since women cannot commit acts of gender violence, no data were obtained in this criminal typology that allowed comparisons with men.

The high percentage of men who claim to commit crimes that cause harm or intend to cause harm (injuries and threats) is noteworthy, while more women claim to commit acts involving fraud, deceit or corruption (see Table 3).

Regarding the criminal variables considered quantitative, results indicated that there were no differences in the sample of men and women studied based on the number of times they were in prison, the age when they were admitted for the first time, sentence time and time served. Significantly longer sentences were observed for men than for women (t = 2.809; gl. = 419; *p* = 0.005 < 0.05). It was not possible to carry out comparative analyses due to the difference in the size of the samples (see Table 4).

### 2.2. Instruments

Physical health was assessed through an item formulated as follows “How would you rate your physical health before entering prison?” with 4 options, where 1 = bad; 2 = average; 3 = good; and 4 = very good. Mental health was measured with a similar item.

Regarding the measurement of anxiety–depressive symptomatology, anxiety was assessed through the State-Trait Anxiety Inventory (STAI) [38] which assesses state and trait anxiety through 40 items (20 for each subscale). Specifically, it was used in its translation into Spanish [39]. The response scale is a Likert-type scale from 0 to 3. In the state anxiety subscale, the response ranges from 0 = not at all, 1 = somewhat, 2 = moderately so to 3 = very much so; meanwhile, in the trait anxiety subscale, the response range is: 0 = almost never, 1 = sometimes, 2 = often and 3 = almost always

Depression symptoms were measured with the Spanish adaptation of the Beck-II Depression Inventory [40], the original instrument being created by Beck, Steer and Brown (1996). The Spanish version has 21 items with four response alternatives.

Finally, the practice of sport in the penitentiary and before entering it, the time spent in prison, if it was your first time in prison, at what age did you enter prison, as well as the sociodemographic data on sex and age, were evaluated by means of an ad hoc questionnaire.

The validity of the information given was due to the attention paid by the research team, which in practical terms involved carrying out an interview instead of applying a questionnaire, by standing next to the inmate and explaining if necessary what the question that was asked meant. What, at first, seemed to be an inconvenience became a very relevant source of information that allowed us to support everything that is narrated here with an exact reflection of reality, as indicated in the following section.

### 2.3. Procedure

In order to access the population under study, it was necessary to make the corresponding permit application to the General Secretariat of Penitentiary Institutions (Ministry of Interior). To formalize this request, among other data, we had to provide all the information related to the research project that we intended to carry out: objectives, lines of research, tools for information collection and temporary planning of field work.

Once the authorization was granted by the Ministry of the Interior, the person in charge of training in the penitentiary was contacted, who was also provided with a presentation letter for its dissemination; this included the objectives of the study, the data to be collected and the informed consent form to be filled in by those people who were willing to collaborate.

The field work was carried out throughout the months of July and August, and the data collection instruments were administered once the training activities with the inmates were completed, respecting the hours of access to the penitentiary. This was carried out in small groups, always following the instructions of the person in charge of training, who called on the different modules to allow access and sent the inmates to the room used for the data collection.

The initial research design contemplated the application of the questionnaire prepared for this purpose and the use of the scales in a group; however, in practice, it had to be performed in a more individualized way, since many inmates constantly needed help to fill it out, which made completion difficult.

The study was approved by the Ethics Committee of the University to which the authors belong and followed the recommendations of the Declaration of Helsinki and the General Data Protection Regulation (2016/679), approved by the European Parliament and the Council of the European Union.

### 2.4. Data Analysis

Data analyses were carried out with the statistical package IBM SPSS Statistics version 25. They were executed sequentially, beginning with the calculation of the descriptive statistics of the variables under study. Then, comparisons of means were made based on sports practice, by comparing groups with Student’s *t*-test. Subsequently, correlation analyses were performed between the different variables of the study using the Pearson correlation coefficient. Finally, a stepwise regression analysis was carried out with the objective of determining to what extent mental health in prison was explained by physical health and anxiety–depressive symptomatology.

## 3. Results

### 3.1. Descriptive Statistics

Table 5 shows the descriptive statistics (mean, standard deviation, minimum and maximum) in the following variables: physical health, mental health, anxiety and depression. The inmates presented moderately high scores in the perception of physical and mental health, both before entering prison and afterwards. The indicators of anxiety and depression were low.

Below, we present the comparisons made in the levels of physical and mental health, before and during their internment, indicated by the inmates, depending on whether or not they practiced sports in prison. Table 6 shows the descriptive statistics of these variables for the two groups: those who practiced sports and those who did not. For this, it was considered that those inmates who indicated doing so at least 3 h a week practiced sports. The distribution of the sample was quite balanced with 41% indicating that they met that standard and 59% indicating that they did more hours of sports.

- Intergroup comparisons before entering prison

There were no significant differences between the group of inmates who practiced sports in prison and the group that did not practice sports prior to imprisonment, both for physical health (t (437) = 0.382; *p* = 0.703) and for mental health (t (434) = 0.882; *p* = 0.378).

- Intergroup comparisons after entering prison

There were significant differences in the levels of physical and mental health after entering prison. The group of inmates who practiced sports in prison had higher levels of physical health (t (248.643) = 7.49; *p* < 0.001; d = 0.81) and mental health (t (434) = 5.009; *p* < 0.001; d = 0.79).

- Intragroup comparisons

**Figure 1:**
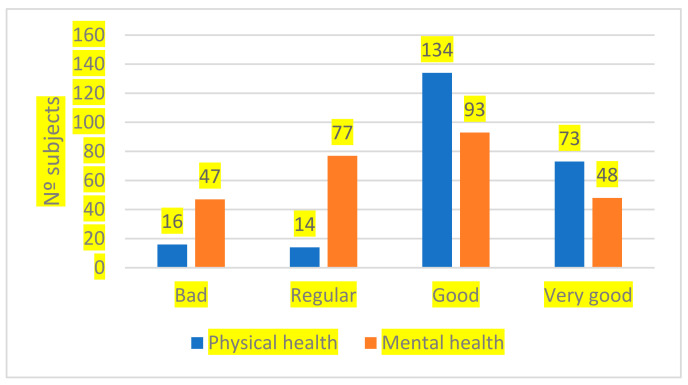
Sports practice in prison and physical and mental health.

Regarding the differences in the levels of physical and mental health within each of the groups, the group of inmates who practiced sports in prison (Figure 1), did not show statistically significant differences between the levels prior to entering prison and the levels shown during imprisonment, both for physical health (t (296) = 1.946; *p* = 0.053) and for mental health (t (296) = 1.958; *p* = 0.051).

On the other hand, the group of inmates who did not practice sports in prison showed a significant decrease in their levels of physical health (t (138) = 5.419; *p* < 0.001) and mental health (t (134) = 4.477; *p* < 0.001).

Regarding the levels of physical and mental health prior to their stay in prison, the group of inmates who practiced sports had higher levels of physical health (M = 2.95; SD = 1) and mental health (M = 2.89; SD = 1.01) than the group of inmates who did not perform any sport, both in physical health indicators (M = 2.45; SD = 1.01) and in mental health indicators (M = 2.56; SD = 1.07). The differences found in relation to physical health (t (432) = 4.973; *p* < 0.001) and mental health (t (331,627) = 3.208; *p* < 0.01; d = 0.83) were statistically significant.

### 3.2. Comparisons in Anxiety and Depression in Those Who Say They Practice Sports and Those Who Do Not

Table 7 shown similar findings to the previous section, the levels of anxiety and depression of the inmates shown in Table 1 were also analyzed according to sports practice. These comparisons can be found in Table 3.

The group of inmates who practiced sports during imprisonment had lower levels of state anxiety, trait anxiety and depression than the group of inmates who did not play sports. These differences are statistically significant.

### 3.3. Correlation Analysis

The correlations among the variables, state anxiety, trait anxiety, depression and physical health in prison and mental health in prison, are shown in Table 4. The dimensions of anxiety and depression correlate with each other positively and significantly. In turn, these variables correlate with physical and mental health in a negative and significant way (see Table 8).

### 3.4. Regression Analysis

The initial model of the multiple linear regression analysis (Table 5) only included, as a predictor variable, physical health with a positive direction. Subsequently, trait anxiety and state anxiety, with a negative direction, were included in a second and third model. The changes in adjusted R^2^ were significant, while the final model explains 49% of the mental health variance. The depression variable was not a significant predictor (see Table 9).

## 4. Discussion

In the present investigation, we wanted to know the degree of physical and mental health as well as anxiety–depressive symptoms in a group of inmates in prisons belonging to the Autonomous Community of Galicia, Spain. We also wanted to know if these levels varied depending on the practice of sports carried out in prison and before their entry into it. In addition, the relationships between these variables were analyzed.

The results indicate that those inmates who performed physical activity in prison showed higher levels of perceived physical and mental health, as well as lower indicators of anxiety and depression. These results are in line with previous scientific literature that has highlighted the benefits of physical activity to improve physical and mental health indicators [2,3,6,18].

The group of inmates who did not perform physical activity in prison worsened their perceived levels of physical and mental health, which did not occur with inmates who did practice sports. These results favor the thesis, widely reinforced by research, that physical inactivity is a risk factor for both physical and mental health [41,42,43].

Regarding the relationships between the variables: physical health, mental health and anxiety–depressive symptoms, the correlation analysis shows a positive relationship between physical health perception and mental health perception. These results are consistent with the scientific literature previously mentioned, as sports performance is substantially associated with mental health [44], a factor that has special importance in the prison population [4,21,45]. The correlation analysis also reflects the existence of negative relationships between the perception of physical health and the perception of mental health with anxiety–depressive symptomatology, as confirmed in previous studies [3,46,47,48,49].

The establishment of the relationships between perceived physical and mental health with anxiety–depressive symptomatology is essential in those inmates diagnosed with a mental disorder, since investigations show how the practice of physical activity also clearly improves their physical and mental health, both in the general population [7] and in prison inmates [19]. Hence the need for research work, perhaps of a more qualitative nature, that allows for enquiries about subjects in the population affected by inherent situations of deprivation, groups that are very difficult to reach with this type of study.

Finally, the results of the regression analysis indicated that the perceived mental health in the inmates is explained to a greater extent by the perceived physical health, which is in line with the existing scientific literature, highlighting the relationship between the attitude towards physical health itself and the perception of mental health [3,50]. In this respect, physical activity is considered as the major factor responsible for the indirect effects between both constructs [51].

Among the limitations of this study, we should mention the impossibility of establishing causal relationships, due to the non-manipulative nature of the investigation, as well as not having measured health through objective indicators, but through questions addressed to inmates about their perceptions of it. However, other previous studies on health perception also evaluate this construct in a similar way [5].

Another limitation has to do with the sample used, which was of an incidental nature, as they were the subjects who voluntarily enrolled in the study. It is considered broad for this type of study; but, even so, it was not balanced by gender because in Galicia there are very few female inmates, and this makes it impossible to establish comparisons based on gender.

## 5. Conclusions

The main conclusion we reached with this study is the difficulty involved in working in prison contexts. A feature of this was the bureaucratic procedures that must be faced from the moment the idea arose, until the permit was obtained; we must add that you were always in the hands of the officials who, as they change daily, you then had to explain again what you were doing there at all hours.

The fact that the inmates have such great mobility meant that you cannot continue working with the same inmate for two weeks in a row, because either he was on trial, on leave, was transferred to another prison or was in a compulsory activity. You have to constantly adapt to the situation.

Each center has its own dynamic of action, which means that different plans had to be used to reach the same end.

The difficulty of working there meant that by having access we tried to reach the majority of inmates and collect the greatest quantity of data, and this can turn against the investigation itself.

This article is a part of a much more complete and complex investigation.

Regarding the subject studied, which seemed very obvious from the outset, it has allowed us to see that there are a multitude of variables that affect it, from the type of center, whether that is more or less crowded, the type of inmates, the prison conditions, the climate that is in it, etc.; all of which may vary the results.

Hence, at this point, it can be concluded that a comparative investigation would be necessary to draw conclusions about the variables that are common and, from there, to investigate each case in depth.

Future lines of research can focus on confirming the present results in the Galician prison population through studies with stratified samples and according to the object of study, which allow for the establishment of a higher degree of causality, more qualitative methodologies and data triangulation.

It would also be important to expand the sample to other Spanish prisons in which there are more women, or to evaluate the levels of physical health through objective indicators.

The studies carried out in recent years are leading to the analysis of cases and groups with a determined casuistry, which may be more effective for progress in the field and contribute to the central objective of achieving the social and labor reintegration of the internees.

## Figures and Tables

**Table 1:** Descriptive statistics of the sample based on criminal variables.

	Men		Women	
	N	%	N	%
First time in prison				
YES	232	52.3	34	56.7
NO	211	47.5	24	40
Several crimes				
YES	108	25.8	4	7.3
NO	311	74.2	51	92.7

**Table 2:** UNODC Level 1 crime categories.

1. Acts leading to death or intending to cause death
2. Acts leading to harm or intending to cause harm to the person
3. Injurious acts of a sexual nature
4. Acts against property involving violence or threat against a person
5. Acts against property only
6. Acts involving controlled psychoactive substances or other drugs
7. Acts involving fraud, deception or corruption
8. Acts against public order, authority and provisions of the State
9. Acts against public safety and state security
10. Acts against the natural environment
11. Other criminal acts not elsewhere classified

**Table 3:** Descriptive statistics of criminal categories according to gender.

	Men		Women	
	N	%	N	%
Acts leading to death or intending to cause death	45	10.9	4	7.5
Acts leading to harm or intending to cause harm to the person	64	15.5	3	5.7
Injurious acts of a sexual nature	11	2.7		
Acts against property involving violence or threat against a person	137	33.1	22	41.5
Acts against property only				
Acts involving controlled psychoactive substances or other drugs	96	23.2	13	24.5
Acts involving fraud, deception or corruption	13	3.1	6	11.3
Acts against public order, authority and provisions of the State	3	0.7	2	3.8
Acts against public safety and state security	2	0.5		
Acts against the natural environment				
Other criminal acts not elsewhere classified	5	1.2	3	5.7
12	38	9.2		

**Table 4:** Descriptive statistics of the quantitative criminal variables according to gender.

	Men		Women	
	N	M (SD)	N	M (SD)
Times in prison	406	1.57 (3.89)	53	1.15 (2.72)
Age when admitted to prison for the first time	409	30.76 (12.01)	49	33.39 (10.99)
Sentence time	371	92.62 (89.41)	50	56.04 (59.42)
Time served (in months)	389	46.95 (58.13)	55	31.05 (45.98)

**Table 5:** Descriptive statistics of the observed variables.

Variable	N	Minimum	Maximum	*M*	*SD*
Physical health before entering prison	447	1	4	2.76	1.04
Mental health before entering prison	444	1	5	2.77	1.04
Physical health in prison	444	1	4	2.67	0.92
Mental health in prison	443	1	5	2.49	0.98
State anxiety	337	0	2.95	1.40	0.64
Trait anxiety	334	0	2.75	1.28	0.54
Depression	341	0	52.00	17.69	10.85

**Table 6:** Descriptive statistics of physical and mental health based on sports practice.

Variable	Practice Sports				Do Not Practice Sports			
	Before Prison		In Prison		Before Prison		In Prison	
	M	SD	M	SD	M	SD	M	SD
Physical health	2.74	1.06	2.89	0.84	2.78	1.01	2.2	0.918
Mental health	2.8	1.03	2.65	0.93	2.7	1.07	2.16	1

**Table 7:** Comparisons in anxiety and depression levels.

Variable	*M (SD)*	*t*	Degrees of Freedom	Bilateral Significance
State anxiety	G1: 1.33 (0.59)	2.258	136.686	0.026
	G2: 1.53 (0.73)			
Trait anxiety	G1: 1.24 (0.53)	2.354	289	0.019
	G2: 1.4 (0.56)			
Depression	G1: 16.69 (10.48)	2.306	301	0.022
	G2: 19.8 (11.43)			

Note G1 = group of inmates who practiced sports during imprisonment; G2 = group of inmates who did not practice sports during imprisonment.

**Table 8:** Correlation analysis.

Variables	1	2	3	4	5
1. State anxiety	1	0.750 **	0.640 **	−0.341 **	−0.522 **
2. Trait anxiety	0.750 **	1	0.726 **	−0.353 **	−0.519 **
3. Depression	0.640 **	0.726 **	1	−0.454 **	−0.543 **
4. Physical health in prison	−0.341 **	−0.353 **	−0.454 **	1	0.591 **
5. Mental health in prison	−0.522 **	−0.519 **	−0.543 **	0.591 **	1

**. The correlation is significant at level 0.01.

**Table 9:** Regression analysis of mental health in prison.

Summary	M1	M2	M3
*Adjusted R^2^*	0.355	0.482	0.49
*F*	113.952 ***	96.435 ***	66.546 ***
Δ*R^2^*		0.129 ***	0.01 *
**Predictor Variable**	***Beta*—M1**	***Beta*—M2**	***Beta*—M3**
Physical health	0.599 ***	0.445 ***	0.426 ***
Trait anxiety		−0.39 ***	−0.281 ***
State anxiety			−0.155 *

Note. M = Model. *. Statistical significance at level 0.05. ***. Statistical significance at level 0.001.

## Data Availability

The data supporting reported results of this article are available from the author (m.rodicio@udc.es) in the Grupo de Investigación FORVI (Formación y Orientación para la Vida), Universidade da Coruña.
